# Microbial physiology and soil CO_2_ efflux after 9 years of soil warming in a temperate forest – no indications for thermal adaptations

**DOI:** 10.1111/gcb.12996

**Published:** 2015-09-28

**Authors:** Andreas Schindlbacher, Jörg Schnecker, Mounir Takriti, Werner Borken, Wolfgang Wanek

**Affiliations:** ^1^ Department of Forest EcologyFederal Research and Training Centre for ForestsNatural Hazards and Landscape ‐ BFWSeckendorff‐Gudent Weg 8A‐2213ViennaAustria; ^2^ Department of Microbiology and Ecosystem ScienceUniversity of ViennaAlthanstraße 14ViennaA‐1090Austria; ^3^ Lancaster Environment CenterUniversity of LancasterLA1 4YQLancasterUK; ^4^ Department of Soil EcologyUniversity of BayreuthDr. Hans‐Frisch‐Straße 1‐3D‐95448BayreuthGermany

**Keywords:** enzyme activities, gross N mineralization, soil CO_2_ efflux, soil warming, substrate use efficiency, thermal adaptation

## Abstract

Thermal adaptations of soil microorganisms could mitigate or facilitate global warming effects on soil organic matter (SOM) decomposition and soil CO_2_ efflux. We incubated soil from warmed and control subplots of a forest soil warming experiment to assess whether 9 years of soil warming affected the rates and the temperature sensitivity of the soil CO_2_ efflux, extracellular enzyme activities, microbial efficiency, and gross N mineralization. Mineral soil (0–10 cm depth) was incubated at temperatures ranging from 3 to 23 °C. No adaptations to long‐term warming were observed regarding the heterotrophic soil CO_2_ efflux (*R*_10_ warmed: 2.31 ± 0.15 μmol m^−2^ s^−1^, control: 2.34 ± 0.29 μmol m^−2^ s^−1^; *Q*_10_ warmed: 2.45 ± 0.06, control: 2.45 ± 0.04). Potential enzyme activities increased with incubation temperature, but the temperature sensitivity of the enzymes did not differ between the warmed and the control soils. The ratio of C : N acquiring enzyme activities was significantly higher in the warmed soil. Microbial biomass‐specific respiration rates increased with incubation temperature, but the rates and the temperature sensitivity (*Q*_10_ warmed: 2.54 ± 0.23, control 2.75 ± 0.17) did not differ between warmed and control soils. Microbial substrate use efficiency (SUE) declined with increasing incubation temperature in both, warmed and control, soils. SUE and its temperature sensitivity (*Q*_10_ warmed: 0.84 ± 0.03, control: 0.88 ± 0.01) did not differ between warmed and control soils either. Gross N mineralization was invariant to incubation temperature and was not affected by long‐term soil warming. Our results indicate that thermal adaptations of the microbial decomposer community are unlikely to occur in C‐rich calcareous temperate forest soils.

## Introduction

Temperature is the primary environmental driver of soil organic matter (SOM) decomposition in temperate ecosystems. Accordingly, there is concern that global warming accelerates the heterotrophic soil CO_2_ efflux (Rh), feeding back to atmospheric CO_2_ concentrations and climate (Cox *et al*., 2000; Friedlingstein *et al*., 2006). The long‐term effect of warming on the soil CO_2_ efflux, however, is unclear as changing SOM availability over time and/or microbial adaptations to warmer conditions affect the temperature sensitivity of Rh and its overall rates. If and to which extent such microbial adaptation mechanisms affect Rh is still a matter of debate (Hartley *et al*., 2007, 2008, 2009; Bradford *et al*., 2008; Allison *et al*., 2010; Vanhala *et al*., 2011; Frey *et al*., 2013; Karhu *et al*., 2014).

Soil warming experiments in the field have proven to be meaningful tools to study the behavior of SOM decomposition and can particularly lead to a better understanding of long‐term warming effects on soil C dynamics. Recent meta‐analyses of *in situ* warming effects showed strong enhancement of soil respiration in global terrestrial ecosystems (Lu *et al*., 2013; Wang *et al*., 2014). However, in some of the longer running manipulation experiments, the initial response to warming declined and diminished over time, suggesting that the warming response of SOM decomposition and soil respiration is transient (Strömgren, 2001; Melillo *et al*., 2002; Lamb *et al*., 2011). Two explanations have been put forward for the observed decreases in soil respiration in continuously warmed field soils: (i) depletion of labile C causes heterotrophic respiration to decline (Melillo *et al*., 2002; Kirschbaum, 2004; Eliasson *et al*., 2005; Hartley *et al*., 2007) and/or (ii) thermal adaptation of microbial processes along with changes in microbial community structure (Luo *et al*., 2001; Bradford *et al*., 2008; Balser & Wixon, 2009; Allison *et al*., 2010; Conant *et al*., 2011; Bradford, 2013). Although the decrease in the warming response could be fully explained by simple soil C pool models (e.g., Kirschbaum, 2004), a simultaneous occurrence of physiological adaptation of the microbial community is concurrent as documented by an array of isolated mycorrhizal fungi (Malcolm *et al*., 2008) and heterotrophic soil microorganisms (Crowther & Bradford, 2013). The significance of such physiological adaptations with regard to the observed response of Rh to warming remains, however, unclear (Rousk *et al*., 2012).

Rh represents a complex process, based on activities of several extracellular enzymes mediating the breakdown of soil organic materials, microbial uptake of labile C compounds, and partitioning of these C sources between microbial growth and respiration. The first step in substrate utilization is the breakdown of complex organic macromolecules by extracellular enzymes which generally responds positively to soil temperature (e.g., Trasar‐Cepeda *et al*., 2007; Wallenstein *et al*., 2009) as long as soil moisture is not limiting (Brzostek *et al*., 2012; Steinweg *et al*., 2012; Suseela *et al*., 2014). The temperature optimum, as well as the temperature‐specific enzyme conformations, can vary between cold‐ and warm‐adapted microorganisms/enzymes (Zavodszky *et al*., 1998; Bradford, 2013). Extracellular enzyme activities can adapt to seasonal fluctuations in soil temperature (Fenner *et al*., 2005; Wallenstein *et al*., 2009; Jing *et al*., 2014) and were found to adapt to local conditions along latitudinal temperature gradients (German *et al*., 2012). Together, this makes potential adaptations of extracellular enzyme kinetics to long‐term soil warming plausible. Once the C‐substrate is taken up by the microbial cell, it is invested in maintenance, growth, or storage. The microbial carbon‐use‐efficiency (CUE; the proportion of C uptake allocated to growth vs. the microbial CO_2_ production by respiration) has been shown to decrease with increasing soil temperature (Steinweg *et al*., 2008; Frey *et al*., 2013; Tucker *et al*., 2013) or to be invariant with temperature (Hopkins *et al*., 2014). Decreased CUE in warmer soils could at least partly explain the declining warming response of Rh over time due to decreasing microbial biomass (Tucker *et al*., 2013). It has also been shown that the temperature sensitivity of CUE depends on the substrate quality and that long‐term warming can lead to the adaptation of microbial physiology toward higher CUE for compounds that are indicators of more stable SOM components (Frey *et al*., 2013).

Adaptations to warming *per se* (‘direct’ thermal adaptations) are often difficult to disentangle from concurrent ‘indirect’ warming effects such as induced declines in SOM availability or quality (Bradford, 2013). In this study, we took advantage of a soil warming experiment in Achenkirch/Austria where soil has been warmed by 4 °C during the snow‐free seasons from 2005 until the current study in 2013. In contrast to other long‐term soil warming experiments, the positive response of soil respiration to warming (~40% increase) remained stable throughout the 9 years of warming (Schindlbacher *et al*., 2012). The sustained enhancement of soil respiration suggests that substrate depletion did not play a major role in this C‐rich soil so far. As the respiration response to warming was sustained over time, we hypothesize (I) that no thermal adaptation of the soil microbial community occurred or that (II) thermal adaptations of microbial processes were not reflected in the soil CO_2_ efflux. In this study, we therefore analyzed the effect of long‐term soil warming on the temperature sensitivity of Rh, extracellular enzyme activities, biomass‐specific respiration rates, microbial substrate use efficiency, and gross N mineralization. Changed temperature sensitivity of these parameters will be considered as thermal adaptation.

## Materials and methods

### Site description and soil sampling

The study site is located at 910 m a.s.l. on a north–north‐east slope of a mountain in the Northern Limestone Alps, Achenkirch, Austria (47°34′50″N; 11°38′21″E). The site is characterized by a cool humid climate. The snow‐free period lasts from April/May to November/December. Local mean annual air temperature and precipitation were 6.9 °C and 1506 mm (1992–2012), respectively [Achenkirch village; ~7 km away at similar altitude; data from Zentralanstalt für Meteorologie und Geodynamik (ZAMG)]. The ~130‐year old forest is dominated by Norway spruce (*Picea abies*), with interspersed European beech (*Fagus sylvatica*) and silver fir (*Abies alba*). The soils are a mosaic of shallow Chromic Cambisols and Rendzic Leptosols. The bedrock is formed of dolomite. Soils are characterized by high carbonate content and near neutral pH. Mull is the dominant humus form and the thickness of the litter + O‐layer reach from 0 to 3 cm. A‐horizons show a strong, small‐scale variability in thickness reaching from 10 to 40 cm. Root density is highest in the O‐ and A‐horizons, and few roots were found to reach down to a depth of 60 cm. Organic C stocks were estimated to be ~10 t ha^−1^ in the organic layer and ~120 t ha^−1^ in the mineral soil (Schindlbacher *et al*., 2010).

In 2004, three experimental plots were randomly selected on the site. Each of the three plots consisted of a warmed and a disturbed‐control subplot (Schindlbacher *et al*., 2009). The subplots had a size of 2 × 2 m each. Warmed subplots were equipped with resistance heating cables (0.4 cm diameter, TECUTE – 0.18 Ohm m^−1^ per UV, Etherma, Austria). The cables were buried in 3‐cm‐deep slots and had a spacing of 7–8 cm. The soil temperature of each warmed subplot was kept 4 °C above that of the adjacent control subplot during the snow‐free seasons, starting in spring 2005. On disturbed‐control subplots, we inserted cables that were not heated, but had inflicted the same soil disturbance as on the warmed subplots.

For the current study, we sampled soil from the warmed (*n* = 3) and the disturbed‐control (*n* = 3, hereinafter ‘control’) subplots. Soil sampling was accomplished during the ninth year of soil warming in early September 2013. We sampled four randomly distributed intact soil cores (diameter 7 cm, depth 10 cm, stainless steel cylinders) from each subplot for the subsequent determination of soil CO_2_ efflux and its temperature sensitivity. We further sampled similar aliquots of mineral soil (0–10 cm) from four randomly distributed spots within each subplot for basic soil parameters and microbial analyses. These mineral soil samples were pooled, mixed, and sieved (2 mm) to obtain a homogenized bulk sample of each subplot for further analytical treatment. Soil was stored in cooling boxes and transported to the laboratory on the day of sampling. Soil temperature at 5 cm depth and field soil CO_2_ efflux were measured on the day of soil sampling from permanently installed chambers, as described in Schindlbacher *et al*. (2012).

### Soil CO_2_ efflux and temperature sensitivity

To assess whether long‐term warming affected the temperature sensitivity of the soil CO_2_ efflux, we simultaneously incubated soil from warmed and control plots under controlled conditions in the laboratory. Undisturbed soil cores, excised roots, and sieved soil were incubated in this order. The incubation was performed with a temperature controlled automatic system as described in Schindlbacher *et al*. (2010, 2004). In brief, an incubator hosted 13 glass chambers (750 cm^3^ each). Twelve chambers were equipped with soil cores, while the 13th chamber was equipped with a stainless steel block of the same size and served as a reference chamber. The system operated as an open‐flow‐through method. Ambient air from inside the incubator was sucked through the chamber headspace to the CO_2_ analyzer (WMA‐4, PP‐Systems, www.ppsystems.com). The CO_2_ efflux from each soil core was calculated from the airflow (1 L min^−1^) and the difference in the headspace CO_2_ concentration of the soil chamber and of the subsequently measured reference chamber (Schindlbacher *et al*., 2008). Chambers were measured consecutively (6 min soil chamber + 2 min reference chamber). A single CO_2_ measuring cycle for all 12 cores lasted ~1.5 h.

Two cores from each plot were immediately placed in the incubator and adjusted to a temperature of 3 °C overnight. The remaining cores were stored in a fridge at 3 °C until incubation. We incubated the undisturbed soil cores at incrementing temperatures from 3 to 23 °C (2.5 °C increments). Incubation at each temperature level lasted for 6 h and allowed for four CO_2_ efflux measurement cycles. Only the last CO_2_ measurement cycle (h 4.5–6) was used for the CO_2_ efflux calculation. Time prior (h 0–4.5) was considered to be for soil temperature increase and for a short equilibration period to the higher temperature. Soil temperature was measured in an additional soil core with a PT100 temperature sensor. The whole incubation procedure lasted for 56 h. The second set of undisturbed soil cores was incubated immediately after the first set was finished (same procedure).

The intact soil cores were disaggregated after the incubation was finished and all the roots were picked out. Fine roots (<2 mm diameter) were washed with water and then placed on wet inert glass fiber mats which were wrapped into the cylinders. To obtain enough root material to establish a measureable CO_2_ efflux, we pooled the roots from the four cores of each subplot. To keep incubation time short, we incubated the excised roots at only five temperatures (3, 8, 13, 18, and 23 °C). As root temperature was considered to equilibrate faster with the incubator temperature, only two CO_2_ measurement cycles were performed at each temperature (3 h) and only the second cycle was used for flux calculation. This allowed finishing the whole root incubation within 15 h.

The root‐free mineral soil of each subplot was homogenized and sieved through a 2‐mm sieve. The homogenized mineral soil from each subplot was repacked into two cylinders at the mean bulk density of the undisturbed soil cores. The sieved soil cores were stored at 3 °C for an equilibration period of ~24 h and were incubated under the same procedure as described above for the undisturbed soil cores.

### Basic soil parameters and microbial biomass

All analyzes described below started 1 day after soil sampling in the field. To determine water content, the sieved mineral soil samples were dried at 80 °C for 48 h. Sieved soils were extracted with 1 m KCl (1 : 7.5 w : v) for 60 min and filtered through ash‐free cellulose filters. Ammonium and nitrate concentrations were determined photometrically following Kandeler & Gerber (1988) and Miranda *et al*. (2001) as modified by Hood‐Nowotny *et al*. (2010), respectively. Total free amino acids (TFAA) were determined fluorometrically following the method described by Jones *et al*. (2002) with modifications by Prommer *et al*. (2014). Microbial C and N were estimated using chloroform fumigation–extraction (Brookes *et al*., 1985) modified after Kaiser *et al*. (2010): Soil samples were fumigated over chloroform for 48 h and were afterward, together with nonfumigated samples, extracted with 1 m KCl solution. Dissolved organic C (DOC) and total dissolved N (TN) were determined in both sets of extracts using a DOC/TN analyzer (Shimadzu TOC‐VCPH/CPN/TNM‐1, Vienna, Austria). Microbial C and N were calculated as the difference between fumigated and nonfumigated samples, without correction for extraction efficiency. DOC, TN, ammonium, nitrate, TFAA, and microbial C and N concentrations were determined in three analytical replicates for each subplot.

### Potential extracellular enzyme activities

Potential enzyme activities were determined fluorometrically and photometrically using a microtiter plate assay (modified after Kaiser *et al*. (2010)). In short, for each subplot three times 1 g of soil was suspended in 100 mL sodium acetate buffer each (100 mm, pH 5.5) and homogenized by ultrasonication. For each sample and each enzyme, 5 wells of a black microtiter plate were filled with 200 μL of the soil slurry. The respective wells were amended with MUF (4‐methylumbelliferyl) labeled substrates: *β*‐D‐glucopyranoside (0.25 mm) for *β*‐glucosidase (BG), *β*‐D‐cellobioside (0.5 mm) for cellobiohydrolase (CBH), and N‐acetyl‐*β*‐D‐glucosaminide (1 mm) for N‐acetyl‐glucosaminidase (NAG). L‐leucine‐7‐amido‐4‐methyl coumarin (1 mm) was used as substrate for leucine aminopeptidase (LAP). Analytical replicated plates for the assays of BG, CBH, NAG, and LAP were incubated simultaneously at five temperatures (3, 8, 13, 18, and 23 °C) for 140 min. Afterward, activity was measured fluorometrically (excitation 365 nm and emission 450 nm). The ratio of C : N acquisition by enzymes was calculated as the sum of the log‐transformed BG and CBH activities divided by the sum of the log‐transformed activities of LAP and NAG. Phenoloxidase (POX) and peroxidase (PEX) activities were measured using L‐3,4‐dihydroxyphenylalanine (DOPA) as substrate in a photometric assay. Three times 1 mL of the original soil slurry was mixed with 1 mL of a 20 mm DOPA solution. After shaking and centrifugation, two wells of each transparent microtiter plates were filled with 250 μL of the supernatant. One of these wells additionally received 10 μL H_2_O_2_ (0.3%) for determination of peroxidase activity. Plates for oxidative enzyme activities were measured photometrically (absorbance 450 nm) at the beginning and after incubation for 20 h at the five temperatures. POX activities were then calculated as the increase in color during the incubation time. PEX activities were calculated as the increase in color during the incubation time from the results of the wells that received H_2_O_2_ minus the results of the wells without H_2_O_2_ addition. Because of condensation on the transparent microtiter plates, which had been incubated at 3 and 8 °C, and which has biased the photometric measurements, only the POX and PEX measurements from the three higher temperatures (13, 18, and 23 °C) were used for further calculations.

### Substrate use efficiency and mass‐specific respiration

To gain an estimate for microbial CUE, we incubated sieved soil samples with a mixture of ^13^C labeled substrates for 24 h and traced ^13^C into CO_2_ and microbial biomass. Such an approach tends to overestimate actual CUE as it does not fully capture microbial growth and maintenance respiration (Sinsabaugh *et al*., 2013). We therefore report our results as substrate use efficiency (SUE).

Samples were incubated with a mixture of uniformly ^13^C‐labeled sugars, amino sugar, organic acids, and amino acids, enriched at 10.4% ^13^C. The overall C : N ratio of the mixture was 20, and the overall degree of reduction, a measure of the chemical energy per unit mole of C, was 4.0. This mixture was chosen to contain low molecular weight compounds available in soils for microbial consumption (Van Hees *et al*., 2005; Manzoni *et al*., 2012). From each subplot and for each temperature (6 subplots × 5 temperatures), soil samples (aliquots of 2 g) were placed in glass bottles (250 mL). The dissolved substrate mixture equivalent to 40 μg C was added to soil samples. The bottles were sealed with butyl rubber plugs (Glasgerätebau Ochs Laborfachhandel e.K., Bovenden, Germany). Using a syringe, 20 mL headspace samples were taken from the bottles and injected into evacuated Exetainers^®^ (Labco Ltd., Ceredigion, UK), directly after adding the ^13^C labeled mixture. The syringe was purged with ambient air between samples. The air removed from the bottles was replaced from a gas bag with known CO_2_ concentration and carbon isotope composition. Samples were incubated at the indicated temperature for 24 h, after which a second set of gas samples was taken. At the end of the incubation period, soil samples were split into equal portions and microbial biomass C (C_mic_) was estimated by chloroform fumigation–extraction. Aliquots of fumigated and nonfumigated K_2_SO_4_ extracts were used to determine *δ* ^13^C of DOC, by direct injection (without column, direct mode) on an HPLC (Dionex Corporation, Sunnyvale, CA, USA) connected through a Finnigan LC‐IsoLink Interface (Thermo Fisher Scientific, Waltham, MA, USA) to a Finnigan Delta V Advantage Mass Spectrometer (Thermo Fisher, Bremen, Germany). *δ* ^13^C signatures of CO_2_ of air samples were analyzed by headspace gas sampler (GasBench II, Thermo Fisher) coupled to an isotope ratio mass spectrometer (Delta V Advantage, Thermo Fisher). CO_2_ reference gas was calibrated using ISO‐TOP gas standards (Air Liquide) with certified ^13^C concentrations. SUE was calculated as follows: SUE = ^13^C_mic_/(^13^C_mic_ + ^13^CO_2_) where ^13^C_mic_ is the substrate ^13^C incorporated into biomass and ^13^CO_2_ is the cumulative substrate ^13^C respired during incubation. Biomass incorporation was calculated as the difference between ^13^C in DOC of chloroform‐fumigated and nonfumigated samples. Cumulative respiration was corrected for the air replaced at the start of the incubation.

Mass‐specific respiration (Rmass) rates were calculated as respiration rates per unit microbial biomass. Rmass was calculated from the SUE incubation data (above). Rh (corrected for the contribution of ^13^C respiration by subtracting the amount of ^13^CO_2_ from total CO_2_) at each incubation temperature (3, 8, 13, 18, and 23 °C) was divided by the microbial biomass content of each sample at the corresponding temperature.

### Gross N mineralization

Gross rates of N mineralization were simultaneously determined for all incubation temperatures (3, 8, 13, 18, and 23 °C) in three replicates per subplot, as described by Kaiser *et al*. (2011). In short, samples were amended with 500 μL of ^15^N labeled (NH_4_)_2_SO_4_ (0.125 mm, 10 at%, Sigma‐Aldrich) in duplicates of 2 g soil, for each temperature. Replicated samples were incubated simultaneously for 4 and 24 h at the respective temperatures and were then extracted with 15 mL of 2 m KCl. NH_4_^+^ was diffused into acid traps which were measured with an elemental analyzer–isotope ratio mass spectrometer (EA‐IRMS) system consisting of a CE Instrument EA 1110 elemental analyzer coupled to a Finnigan MAT Delta^Plus^ IRMS with a Finnigan MAT ConFlo II Interface. Gross rates were calculated as described by Wanek *et al*. (2010).

### Data analysis

The temperature response of the soil core CO_2_ efflux, of enzyme activities, of gross N mineralization, and of SUE was determined by means of an exponential *Q*_10_ function (Janssens & Pilegaard, 2003):

(1) $$R=R_{10}*Q_{10}^{\left((T-10)/10\right)}$$

in which *R* is the measured process rate (soil CO_2_ efflux, gross N mineralization, Rmass, SUE, ^13^C incorporation, and ^13^C respiration), *R*_10_ is the simulated process rate at 10 °C, *Q*_10_ is the temperature sensitivity of the process rate, and *T*, the independent variable, is the soil temperature. The *R*_10_ and *Q*_10_ were fitted to the measured *R* and temperature data by means of a nonlinear least square fitter (SigmaPlot for Windows, version 12.0; SysStat Software, Inc., San Jose, CA, USA).

*Q*_10_ and *R*_10_ values were calculated for each individual subplot; that is, the *Q*_10_ function was fitted to the mean CO_2_ efflux of the replicated soil cores from each subplot (intact cores: 4 replicates per subplot; sieved soil: 2 replicates; roots: 1 replicate). *Q*_10_ and *R*_10_ values were calculated for each subplot of the control and warming treatment for statistical analysis. Mean *Q*_10_ and *R*_10_ of CO_2_ efflux from undisturbed cores, roots, and sieved soil cores (*n* = 3) were statistically analyzed using a *t*‐test (SigmaPlot for Windows, version 12.5, SysStat Software, Inc.).

*Q*_10_ and *R*_10_ of enzyme activities, gross N mineralization, SUE, ^13^C incorporation as well as ^13^C respiration were statistically analyzed using *t*‐tests as well (SigmaPlot for Windows, version 12.5, SysStat Software, Inc.). In addition, the effects of incubation temperature during the experiment and of soil warming *in situ* (treatment effect), as well as their interaction, were tested by two‐way anova, after checking for homogeneity of variance using the Levene test. anovas were calculated using Statgraphics Centurion XVI software (StatPoint Technologies, Inc., Warrenton, VA, USA).

## Results

**Figure 1:**
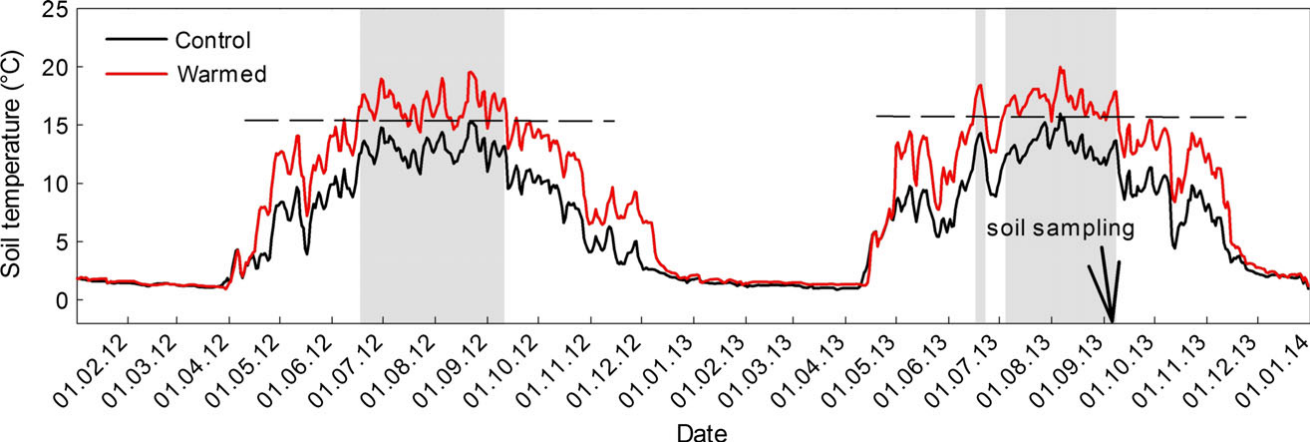
Daily mean temperatures at 5 cm soil depth at control and warmed subplots during 2012 and 2013. Soil was not warmed during snow cover. Gray bars indicate periods during which the warmed subplot temperatures exceeded the maximum control subplot temperature of the corresponding year (dashed line). The arrow indicates the date of soil sampling for incubation.

2012 and 2013 soil temperatures at warmed and control subplots are presented in Fig. 1. Mean soil temperatures at 5 cm depth during soil sampling (03 Sept 2013 12:00) were 15.6 ± 0.2 °C at the warmed and 11.6 ± 0.4 °C at the control subplots. Corresponding soil temperatures at 15 cm depth were 10.2 ± 0.3 °C at the warmed and 13.4 ± 0.3 °C at the control subplots. Mean field soil CO_2_ efflux at 03 September 2013 was 6.2 ± 1.1 μmol m^−2^ s^−1^ at the warmed subplots and 4.1 ± 0.6 μmol m^−2^ s^−1^ at the control subplots (not shown). Soils at both, warmed and control, subplots showed high soil moisture contents (Table 1). All basic soil parameters showed a trend (though nonsignificant) toward lower values in the warmed subplots (Table 1).

**Table 1:** Basic mineral soil (0–10 cm soil depth, means ± SE, *n* = 3) parameters from warmed and control subplots. Samples were obtained during the ninth year of soil warming. n.s. not significant

	Control	Warmed	*t*‐Test
Water content (% fresh soil)	48.3 ± 3.8	44.1 ± 2.5	n.s.
Extractable organic C (μg C g^−1^ dw)	123 ± 6.5	104 ± 6.8	n.s.
Extractable N (μg N g^−1^ dw)	29.8 ± 1.5	24.7 ± 1.86	n.s.
Ammonium (μg N g^−1^ dw)	2.17 ± 0.17	2.44 ± 0.37	n.s.
Nitrate (μg N g^−1^ dw)	4.44 ± 0.32	3.07 ± 0.51	n.s.
Total free amino acids (μg N g^−1^ dw)	2.31 ± 0.10	2.15 ± 0.13	n.s.
Microbial C (μg C g^−1^ dw)	847 ± 143	696 ± 25	n.s.
Microbial N (μg N g^−1^ dw)	182 ± 31	154 ± 5	n.s.

**Figure 2:**
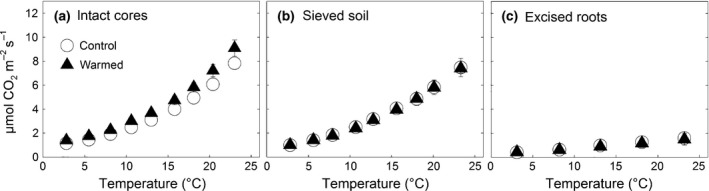
Effect of increasing soil temperature on the CO_2_ efflux from (a) intact soil cores, (b) cores packed with sieved mineral soil, and (c) the roots which were excised during the disaggregation of the intact soil cores. Soil cores (0–10 cm soil depth) were sampled from subplots which had been exposed to soil warming throughout 9 seasons (full triangles, means ± SE, *n* = 3) and from subplots which had experienced the corresponding natural temperature conditions (open circles, means ± SE, *n* = 3).

CO_2_ efflux from all incubated soil cores increased exponentially with increasing incubation temperature (Fig. 2). The *Q*_10_ function showed an excellent fit for CO_2_ effluxes of all undisturbed cores (*R* ^2^ 0.995–0.999) and the sieved soil cores (*R* ^2^ 0.993–0.999). The fit of the root CO_2_ effluxes was weaker but still highly significant (*R* ^2^ 0.913–0.997). The mean CO_2_ efflux of the intact soil cores from the warmed subplots was slightly higher as the CO_2_ efflux of the control subplot cores across all incubation temperatures (Fig. 2), but the difference in *R*_10_ was not statistically significant (Table 2). The temperature sensitivity of soil CO_2_ efflux (*Q*_10_) from intact cores did not differ significantly between warmed and control subplots (Table 2). Mean mass of fine roots was similar in warmed subplot root cores (5.5 ± 1.5 g d w core^−1^) and control subplot root cores (5.7 ± 0.7 g d w core^−1^) as was the root CO_2_ efflux (Fig. 2) and its temperature sensitivity (Table 2). Sieved soil core CO_2_ efflux rates and their temperature sensitivity were similar for both, warmed and control, subplots at all incubation temperatures (Fig. 2) producing correspondingly similar *R*_10_ and *Q*_10_ values (Table 2).

**Table 2:** Mean *Q*_10_ and *R*_10_ values (± SE, *n* = 3) of soil respiration rates, extracellular enzyme activities [*β*‐glucosidase (BG), cellobiohydrolase (CBH), N‐acetyl‐glucosaminidase (NAG), leucine aminopeptidase (LAP), phenoloxidase (POX), and peroxidase (PEX)], substrate use efficiency and its components, mass‐specific respiration (Rmass), and gross N mineralization. Soil was incubated at temperatures ranging from 3 to 23 °C. (n.s. not significant; n.a. not analyzed)

Process	*Q*_10_			*R*_10_		
	Control	Warmed	*t*‐Test	Control	Warmed	*t*‐Test
Soil respiration						
Intact soil cores (μmol CO_2_ m^−2^ s^−1^)	2.56 ± 0.04	2.52 ± 0.06	n.s.	2.30 ± 0.11	2.77 ± 0.23	n.s.
Sieved soil cores (μmol CO_2_ m^−2^ s^−1^)	2.45 ± 0.04	2.45 ± 0.06	n.s.	2.34 ± 0.29	2.31 ± 0.15	n.s.
Roots (μmol CO_2_ m^−2^ s^−1^)	1.85 ± 0.02	1.81 ± 0.05	n.s.	0.73 ± 0.02	0.73 ± 0.24	n.s.
Extracellular enzyme activities						
BG (pmol g^−1^ dry soil s^−1^)	2.06 ± 0.06	1.95 ± 0.18	n.s.	12.6 ± 1.5	17.3 ± 0.8	n.s.
CBH (pmol g^−1^ dry soil s^−1^)	1.46 ± 0.04	1.49 ± 0.08	n.s.	66.0 ± 5.5	71.4 ± 4.7	n.s.
NAG (pmol g^−1^ dry soil s^−1^)	1.48 ± 0.07	1.56 ± 0.13	n.s.	172 ± 18.3	142 ± 8.9	n.s.
LAP (pmol g^−1^ dry soil s^−1^)	1.61 ± 0.03	1.66 ± 0.05	n.s.	33.7 ± 4.0	27.2 ± 1.0	n.s.
POX (pmol g^−1^ dry soil s^−1^)	4.77 ± 0.24	4.21 ± 0.32	n.s.	20.1 ± 7.0	16.6 ± 1.5	n.s.
PEX (pmol g^−1^ dry soil s^−1^)	2.82 ± 0.19	2.85 ± 0.10	n.s.	101 ± 29.4	78.5 ± 5.3	n.s.
Ratio of C : N acquisition	n.s.	n.s.	n.a.	n.s.	n.s.	n.a.
Substrate use efficiency						
^13^C respiration (pmol C g^−1^ dry soil s^−1^)	1.47 ± 0.04	1.47 ± 0.02	n.s.	11.2 ± 0.7	10.2 ± 0.8	n.s.
^13^C incorp. (pmol C g^−1^ dry soil s^−1^)	n.s.	n.s.	n.a.	n.s.	n.s.	n.a.
Substrate use efficiency	0.88 ± 0.01	0.84 ± 0.03	n.s.	0.72 ± 0.02	0.74 ± 0.01	n.s.
Microbial C (μmol g^−1^ dry soil)	n.s.	n.s.	n.a.	n.s.	n.s.	n.a.
SOM respiration (pmol C g^−1^ dry soil s^−1^)	2.75 ± 0.17	2.54 ± 0.23	n.s.	45.3 ± 7.83	42.4 ± 10.1	n.s.
Rmass (nmol C g^−1^ Mic C s^−1^)	2.62 ± 0.25	2.45 ± 0.19	n.s.	37.1 ± 5.55	42.0 ± 11.4	n.s.
Gross nitrogen processes						
N mineralization	n.s.	n.s.	n.a.	n.s.	n.s.	n.a.

**Figure 3:**
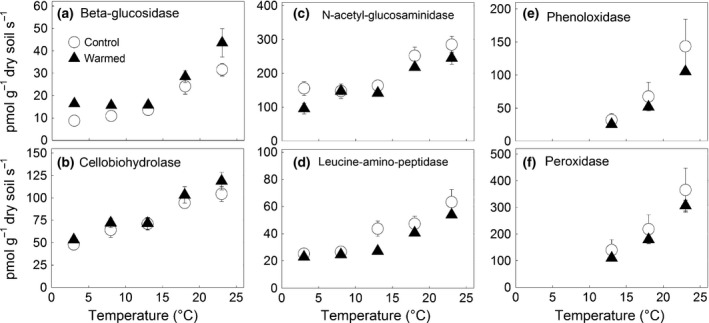
Temperature effects on the extracellular enzyme activities of the mineral top‐soil ((a) *β*‐glucosidase, (b) cellobiohydrolase, (c) N‐acetyl‐glucosaminidase, (d) leucine aminopeptidase, (e) phenoloxidase, and (f) peroxidase) from subplots which had been exposed to soil warming throughout 9 seasons (full triangles, means ± SE, *n* = 3) and from subplots which had experienced the corresponding natural temperature conditions (open circles, means ± SE, *n* = 3). Mineral soil from each subplot was incubated simultaneously at the indicated temperatures.

**Figure 4:**
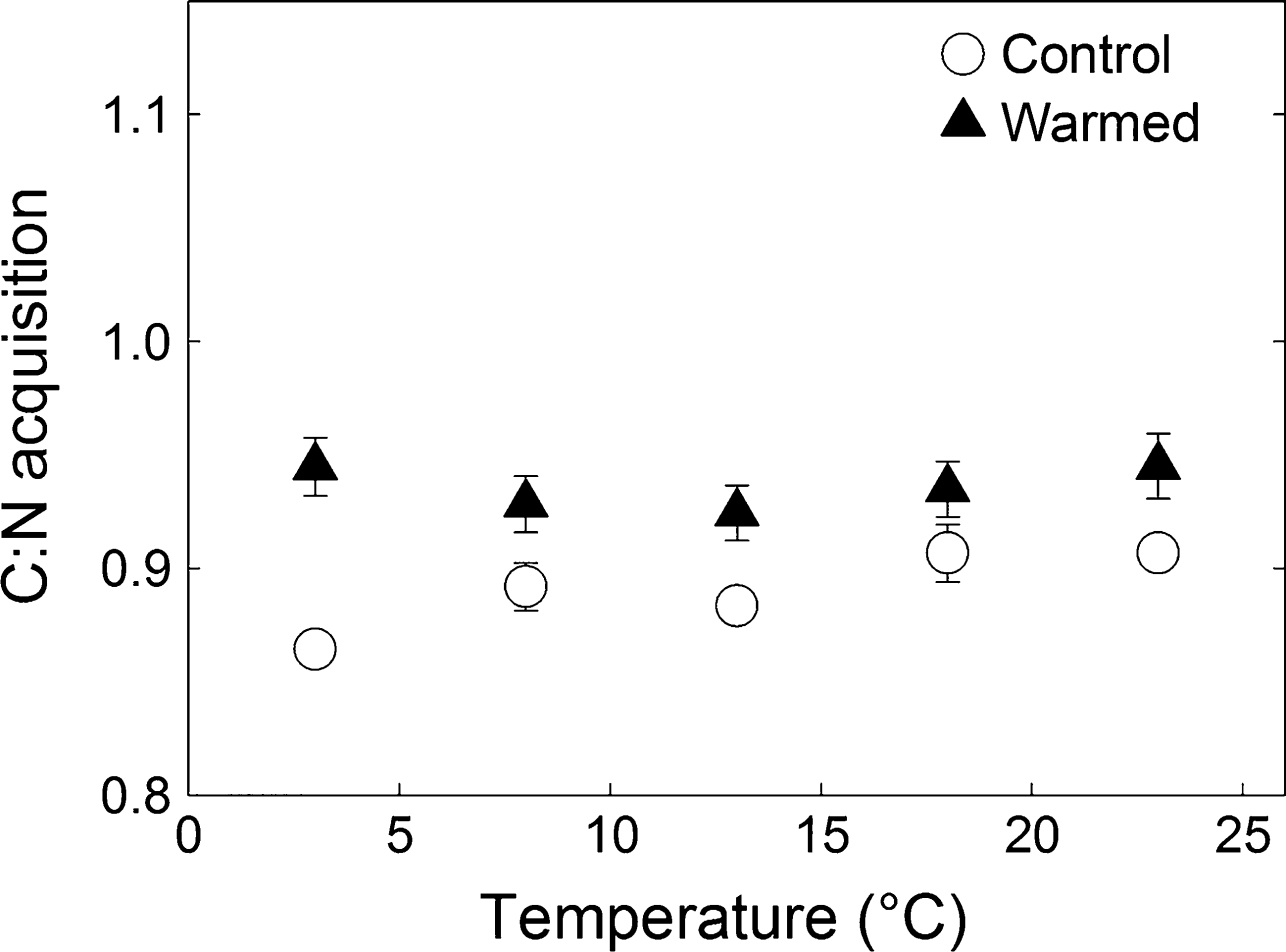
Ratio of enzymatic C : N acquisition, calculated as the sum of the log‐transformed activities of beta‐glucosidase and cellobiohydrolase activity over the sum the log‐ transformed activities of N‐acetyl‐glucosaminidase and leucine aminopeptidase activity at different incubation temperatures. Full triangles represent the values for soil which was exposed to 9 years of warming (means ± SE, *n* = 3). Open circles represent the values for soil which had experienced the corresponding natural temperature conditions (means ± SE, *n* = 3).

Potential extracellular enzyme activities all showed a significant positive relationship with incubation temperature (Fig. 3, Tables 2 and 3). The model fit was weaker than for soil respiration (*R* ^2^ 0.686–0.999) but significant in all cases (*P* < 0.05). *Q*_10_ and *R*_10_ values of potential enzyme activities did not differ significantly for any enzyme between the treatments (Table 2). The temperature sensitivity of the different individual enzymes varied substantially (*Q*_10_ 1.5–4.8), the oxidative enzymes being the most sensitive to soil temperature. According to two‐way anova, BG activity was significantly higher in warmed subplot soils, whereas NAG and LAP activities were significantly lower in warmed subplot soils (Table 3). The ratio of C : N acquisition, calculated as the sum of BG and CBH over the sum of NAG and LAP, showed no increase with incubation temperature, but was significantly higher in soils from warmed subplots (Table 3, Fig. 4).

**Table 3:** Results of two‐way anova analysis of the factors soil warming treatment and incubation temperature and their interaction on soil respiratory processes, extracellular enzyme activities, microbial substrate use efficiency and its components, mass‐specific respiration (Rmass), and gross N mineralization. Variance homogeneity was checked by Levene's test and was given for all data. Significant effects are highlighted in bold

Process	Warming treatment		Incubation temperature		Interaction	
	*F*	*P*	*F*	*P*	*F*	*P*
Soil respiratory processes						
Intact soil cores	6.3	**<0.001**	35.9	**<0.001**	0.2	0.886
Sieved soil cores	0.1	0.873	49.6	**<0.001**	0.0	1.000
Root respiration	0.1	0.838	4.9	**0.007**	0.0	0.999
Soil dry mass‐based/enzyme activities						
*β*‐Glucosidase	8.5	**0.009**	20.6	**<0.001**	0.6	0.650
Cellobiohydrolase	2.0	0.177	17.2	**<0.001**	0.2	0.944
Chitinase	5.2	**0.034**	16.1	**<0.001**	0.5	0.711
Leucine aminopeptidase	5.3	**0.033**	15.9	**<0.001**	0.7	0.609
Phenoloxidase	1.1	0.318	8.5	**0.005**	0.2	0.792
Peroxidase	0.9	0.360	7.7	**0.007**	0.0	0.965
Ratio of C : N acquisition	29.8	**<0.001**	1.2	0.360	1.2	0.327
Substrate use efficiency						
Microbial C	3.2	0.088	0.15	0.962	0.1	0.977
^13^C‐respiration	2.5	0.131	21.2	**<0.001**	0.1	0.974
^13^C‐incorporation	3.0	0.099	0.5	0.763	0.3	0.862
Substrate use efficiency	0.1	0.806	9.6	**<0.001**	0.3	0.857
SOM respiration	1.2	0.297	20.6	**<0.001**	0.4	0.805
Rmass	0.1	0.829	18.7	**<0.001**	0.1	0.965
Gross N processes						
N mineralization	0.5	0.492	0.3	0.851	0.1	0.973

**Figure 5:**
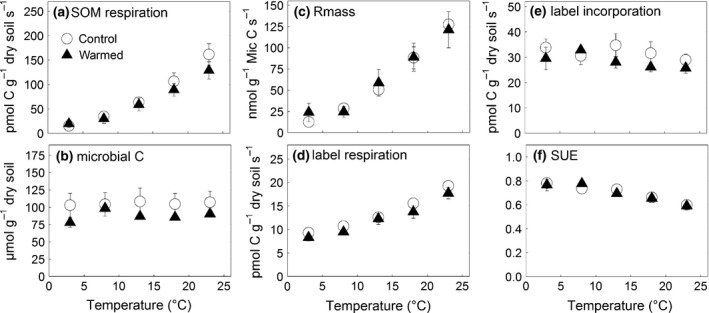
Effects of soil temperature on decomposer efficiency. Heterotrophic respiration of SOM, corrected for ^13^C respiration (a); microbial biomass C (b); mass‐specific respiration (c); respired ^13^C label (d); ^13^C label incorporated into microbial biomass (e); and substrate use efficiency (SUE, f). Full triangles represent the values from long‐term warmed soil (means ± SE, *n* = 3). Open circles represent the values of soil which had experienced the corresponding natural temperature conditions (means ± SE, *n* = 3). Mineral soil from each subplot was incubated simultaneously at the indicated soil temperatures for 24 h.

Microbial SUE did not differ between soils from warmed and control subplots (Table 3). For both treatments, ^13^C respiration increased exponentially with increasing incubation temperature, whereas ^13^C incorporation into the microbial biomass showed no response to incubation temperature (Fig. 5). Accordingly, microbial SUE decreased with increasing incubation temperature (Fig. 5). There was no significant field warming effect on the rates of ^13^C incorporation or ^13^C respiration (Table 3). The temperature sensitivity of microbial SUE did not differ between warmed and untreated soils either (Tables 2 and 3).

Rmass increased exponentially with incubation temperature (Fig. 5, Table 3) as microbial biomass C was unaffected by incubation temperature and the respiration corrected for the ^13^C label (SOM respiration) increased in a similar manner as observed for the undisturbed cores incubation (Tables 2 and 3). Long‐term field soil warming did not affect the temperature sensitivity of Rmass (Table 2).

**Figure 6:**
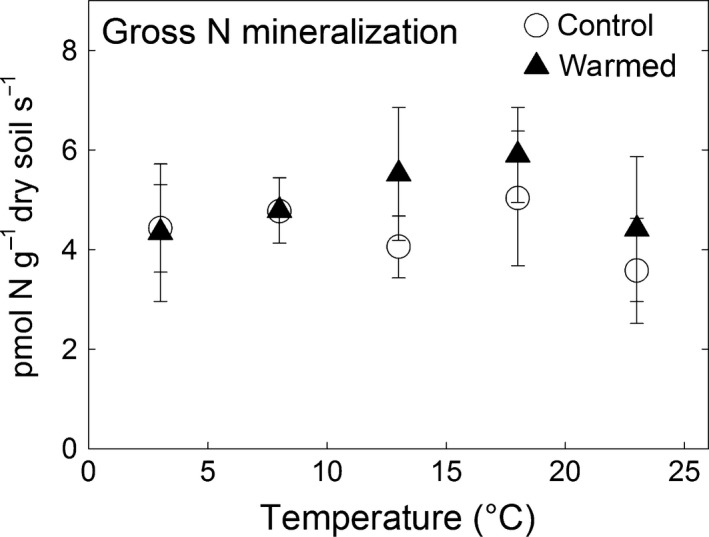
Effect of soil temperature on gross N mineralization. Full triangles represent the values from long‐term warmed soil (means ± SE, *n* = 3). Open circles represent the values of soil which had experienced the corresponding natural temperature conditions (means ± SE, *n* = 3). Mineral soil from each subplot was incubated simultaneously at the indicated soil temperatures.

Gross N mineralization showed, in contrast to respiration and enzyme activities, no increase with incubation temperature (Table 3, Fig. 6) and was not affected by long‐term warming (Table 3).

## Discussion

Thermal adaptation of microbial processes could strongly impact future soil C stocks and atmospheric CO_2_ concentrations by accelerating or mitigating the response of Rh to global warming (Wieder *et al*., 2013; Hagerty *et al*., 2014; Karhu *et al*., 2014), but our results indicate that such thermal adaptations are, at least for the studied soil type, unlikely to occur.

Rh as well as the temperature sensitivity of Rh did not differ between the soil which was warmed during 9 consecutive growing seasons and the soil which had experienced natural temperature conditions when soil was incubated under controlled conditions. This suggests that no thermal adaptations of the decomposer community had occurred as of yet. The sustained strong response of the soil CO_2_ efflux to field warming (~50% higher CO_2_ efflux at warmed subplots during the day of soil sampling), and the almost equal CO_2_ efflux from the incubated sieved soil indicate that ‘indirect’ effects such as substrate limitation had not affected the decomposer community in the studied soil until now. With the consecutive incubation of intact soil cores, roots, and sieved soil, we aimed to account for potential effects of soil disruption on the temperature sensitivity of Rh during the incubation. As the temperature sensitivity of Rh is influenced by SOM quality/recalcitrance (Conant *et al*., 2008; Karhu *et al*., 2010), disruption of soil aggregates and a release of easily decomposable SOM during soil sieving could alter Rh rates and their temperature sensitivity. Such a disruption effect can, however, be neglected in our study as the *Q*_10_ values from intact soil cores and sieved soil were similar and showed the same pattern when compared to long‐term soil warming. Fine root amount, root respiration rates, and their temperature sensitivity were similar in cores from warmed and control subplots. Therefore, with regard to Rh, the comparability of the intact soil core CO_2_ efflux from warmed and control subplots was given as well. The slightly higher soil CO_2_ efflux of the intact cores from the warmed plots indicates a higher availability of labile C at the warmed plots, which might, at least partly, be explained by increased fine root turnover due to long‐term soil warming (A. Schindlbacher, unpublished data). Neither the low contribution of root derived CO_2_ to the soil core efflux, nor the temperature sensitivity of the CO_2_ efflux of cutoff roots should be considered representative of field conditions because roots were cut off several days prior to the incubation.

Although the uniform response of Rh to increasing soil temperature indicates the absence of thermal adaptations, it is possible that individual microbial processes have adapted to the long‐term warming, but that individual adaptations outbalanced each other. We therefore separately assessed a number of underlying C cycling processes. The first step in microbial substrate and nutrient acquisition is the production and release of extracellular enzymes which break down complex organic macromolecules and provide soluble low molecular weight products for microbial uptake and assimilation. The effect of long‐term soil warming on extracellular enzyme activities has, however, rarely been studied in the field. In accordance with Allison & Treseder (2008), Jing *et al*. (2014), Steinweg *et al*. (2013), and Weedon *et al*. (2014), we could not find a clear response of enzyme activities to the long‐term soil warming. Like us, Allison & Treseder (2008) observed slightly lower NAP activity in an artificially warmed boreal forest soil. Other enzyme activities were not affected by warming in their study. We observed higher BG activity in warmed soil together with a constantly higher ratio of enzymatic C : N acquisition. This indicates that the microbial communities in the warmed soil had shifted their energy investments toward C acquisition. The enhanced investment in C‐acquiring enzymes could be a strategy to cope with the increased temperature and the concomitant higher demand for C for respiration (discussed below). In accordance with Jing *et al*. (2014), we did not observe any thermal adaptation in the temperature sensitivity of the enzyme activities. The study of Jing *et al*. (2014) is based on seasonal changes in enzyme activities, whereas we conducted a point in time assessment. As the extracellular enzyme activity can adapt seasonally (Wallenstein *et al*., 2009), it cannot be excluded that thermal adaptations in extracellular enzyme kinetics had occurred during other seasons than during soil sampling in our study. The likelihood of such adaptations, however, is low as the microbial community composition at our site experiences only minor seasonal variations and was not affected by soil warming (Schindlbacher *et al*., 2011; Kuffner *et al*., 2012).

Extracellular enzymes provide the compounds that can be assimilated by soil microorganisms. The CO_2_ of aerobic respiration is produced within the microbial cells and the heterotrophic soil CO_2_ efflux therefore results from intracellular reactions. Therefore, trade‐offs between microbial energy investments in different growth and maintenance strategies affect the decomposition (and formation) of SOM and correspondingly determine Rh rates (Bradford, 2013). Rmass gives a first clue about potential adaptations in microbial efficiency. Rmass increased with incubation temperature but was not different in long‐term warmed soil and control soil. Accordingly, thermal adaptations regarding Rmass did not occur in our long‐term warming experiment. Our Rmass values may hold some uncertainty as the 24‐h incubation period might not have been sufficiently long enough to allow for full equilibration of microbial biomass to the different temperature regimes. However, we periodically assessed microbial biomass in the field from 2008 until 2010 (4th–6th year of warming) and found that warming had not affected microbial biomass C, while the soil CO_2_ efflux was substantially increased (Schindlbacher *et al*., 2011). Therefore, warming had persistently enhanced Rmass in the field, confirming our incubation results.

In contrast to Rmass, microbial SUE decreased with increasing incubation temperature, indicating that some energy demanding processes outbalanced the microbial C gain at increasing temperatures. The increase in ^13^C respiration (Fig. 3) suggests that primarily maintenance energy costs increased with temperature (Manzoni *et al*., 2012). The decrease in microbial SUE of ~0.009 °C^−1^ is consistent with the estimate of 0.009 °C^−1^ derived by Steinweg *et al*. (2008) using a similar approach and with the decrease of microbial CUE by 0.011‐ 0.017 °C^−1^ in the study of Tucker *et al*. (2013). Frey *et al*. (2013) did not observe a negative response of microbial CUE to soil temperature for glucose but a strong negative response of more recalcitrant compounds (glutamic acid – 0.9 °C^−1^, phenol – 1.1 °C^−1^), whereas Hagerty *et al*. (2014) did not observe any temperature response. The frequently observed negative relationship between microbial CUE and soil temperature raised the question whether this relationship could be a cause of thermal acclimation of soil respiration (Tucker *et al*., 2013). Our results do not support this hypothesis. The temperature sensitivity of microbial SUE was, however, similar. Hagerty *et al*. (2014) proposed that the apparent decline in microbial CUE at higher soil temperatures is actually caused by increased microbial turnover rates. This would, however, not have affected our estimates of microbial SUE as this was assessed only over a 24‐h period. Applying their higher microbial turnover scenario, the model predicts a strong decrease in the microbial biomass pool in the longer term (Hagerty *et al*., 2014). Such a decrease in microbial biomass did not occur in our field experiment so far.

The increase in enzymatic C : N acquisition ratios in warmed soils points to possible impacts on the soil N cycle. However, we found no thermal adaptation of gross N mineralization in the long‐term warming treatment, similar as reported in a recent meta‐analysis of the few explicit studies available on this subject (Bai *et al*., 2013). Surprisingly, gross N mineralization showed no temperature sensitivity in our incubation study which concurs with results found for managed temperate soils (Lang *et al*., 2010) but contrasts with findings from others where gross N mineralization increased with incubation temperature (Binkley *et al*., 1994; Cookson *et al*., 2002, 2007; Huber *et al*., 2007; Schütt *et al*., 2014). Gross N mineralization is driven by the rate of organic N release in soils (and microbial organic N uptake) and the microbial nitrogen‐use efficiency (NUE) (Mooshammer *et al*., 2014). Similar to CUE, microbial NUE depicts how much of the organic N taken up is incorporated into microbial biomass, the excess being excreted as ammonium thereby fueling gross N mineralization (Mooshammer *et al*., 2014). The results observed here indicate that organic N release through extracellular enzymes increased with temperature (see NAG and LAP). The absence of short‐term stimulation of gross N mineralization by temperature can therefore be reconciled by increasing microbial NUE with temperature which lowers the fraction of organic N taken up by microbes that is mineralized. The proposed increase in microbial NUE could possibly be triggered by the increase in the release of organic C relative to organic N with temperature, causing an increased microbial N demand (or N limitation).

As thermal adaptations were documented for a variety of decomposer microorganisms, the question remains why microbial traits did not adapt to elevated soil temperature in our study. The answer may lie in the heterogeneity of the microbial community (Kuffner *et al*., 2012) but probably also in the strong seasonal and diurnal temperature fluctuations of this ecosystem. Field soil temperatures fluctuate daily, seasonally, and interannually (Fig. 1). This implies that soil in unwarmed subplots experienced, with some time lag, similar temperatures as the warmed subplots. Only during summer, warmed subplot temperatures actually exceed the temperatures which are experienced by the control soils (Fig. 1). Therefore, it should be questioned, if, and to which extent physiological adaptations to higher or lower temperatures, which were typically observed under rather static temperature conditions, apply in ecosystems which are characterized by recurrent and strong temperature fluctuations (Wallenstein & Hall, 2012).

We did not warm the soil during the dormant season because it would eliminate the isolating snow cover. In subalpine and alpine climate zones, the consequences of warmer air temperatures for soil temperatures during winter rely on the existence and condition of the snow cover. In the study region, climate warming will reduce the duration of snow cover with a general trend toward warmer soils during wintertime (Kreyling & Henry, 2011). However, a missing snow cover can also cause severe soil frost during cold winters with respective implications for soil C cycling (Groffman *et al*., 2001; Muhr *et al*., 2009). At our study site, soil temperature might be not or less affected at future elevated winter air temperatures as long as snow covers the soil surface.

Our data show that even 9 years of intensive soil warming do not necessarily lead to a thermal adaptation of microbial processes. Reasons for this might be that the investigated soil, with its high SOM content, provided the C to fulfill the higher microbial energy demand at the higher temperatures, but also the nutrients to produce the enzymes that were necessary to access additional C sources. This may change when warming reduces the substrate pool to a level at which microbes become limited in C supply. Limited substrate availability can alter the microbial community structure (Frey *et al*., 2008; DeAngelis *et al*., 2015) and function (Bradford *et al*., 2008). If and how such ‘microbial community response’ (Karhu *et al*., 2014) affects future soil C cycling, however, also depends on the response of the above‐ and belowground biomass to global change (Bronson *et al*., 2008). Increased plant productivity and C input into the soil could retain the availability of SOM. Our results indicate that under such circumstances, a thermal adaptation of microbial physiology to higher soil temperatures seems unlikely. Therefore, global warming could cause substantial C losses from C‐rich temperate forest soils to the atmosphere and thereby offset the predicted increase of C inputs to soils from vegetation. Microbial physiology can adapt when soils experience temperatures above the optimum conditions for microbial growth and C mineralization (Bárcenas‐Moreno *et al*., 2009; Birgander *et al*., 2013). At our site, however, soil temperatures in the warming plots were far below the optimum temperatures for SOM decomposition.
